# Effects of 16 Weeks of Cheerleading on Physical Self-Esteem and Mental Health of Female College Students

**DOI:** 10.3389/fpsyg.2022.925162

**Published:** 2022-06-10

**Authors:** Chenliang Deng, Qiaoyan Yu, Ganglin Luo, Shuiping Lu

**Affiliations:** ^1^Sports Department, University of Electronic Science and Technology of China, Chengdu, China; ^2^School of Gymnastics, Chengdu Sport University, Chengdu, China; ^3^Department of Military Education and Training, Police Academy of the Armed Police, Chengdu, China; ^4^Department of Physical Education, Jinjiang College of Sichuan University, Meishan, China

**Keywords:** cheerleading, female college students, physical self-esteem, mental health, intervention effect

## Abstract

**Objective:**

This study aimed to analyze the influence of cheerleading on female college students’ physical self-esteem and mental health.

**Materials and Methods:**

In total, 63 female college students from the University of Electronic Science and Technology of China were trained in cheerleading for 16 weeks. The scores of each sub-field of physical self-esteem and psychological symptoms were analyzed by using Physical Self-Perception Profile (PSPP) and Symptom Checklist 90 (SCL-90), respectively, at 0 and 16 weeks of the experiment.

**Results:**

After 16 weeks of cheerleading exercise, female college students had significant differences in physical attractiveness (*T* = 4.18), physical quality (*T* = 4.39), and physical self-worth (*T* = 3.35) before and after the experiment (*P* < 0.01). There were significant differences in physical condition (*T* = 3.87) and athletic ability (*T* = 2.88) before and after the experiment (*P* < 0.05). Somatization (*T* = 6.485), obsessive-compulsive symptoms (*T* = 11.716), interpersonal sensitivity (*T* = 10.077), depression (*T* = 8.403), anxiety (*T* = 10.767), hostility (*T* = 10.866), terror (*T* = 9.260), paranoia (*T* = 10.414), psychosis (*T* = 9.610), sleep and eating disorders (*T* = 9.323), total symptom index (*T* = 13.245), and mean score of positive symptoms (*T* = 12.237) were significantly different (*P* < 0.01).

**Conclusion:**

Cheerleading can significantly improve the level of female college students’ physical self-esteem, especially the self-esteem level of physical attractiveness, physical quality, and physical self-worth. They also experienced significant improvement in their psychological disorders, especially somatization, depression, and sleep and eating disorders, which effectively improved their overall mental health.

## Introduction

Physical self-esteem refers to individual satisfaction or dissatisfaction with different aspects of the body that are closely related to social evaluation (Chen, 2003). Including sports ability, physical condition, physical attraction, physical quality, and physical self-worth, it can fully reflect the ante-cause and psychological effect of individual participation in physical exercise (Cai, 2007). With the rapid development of society and the continuous improvement of living standards, female college students pay more attention to their body shape than other groups, and their physical self-esteem consciousness is gradually enhanced with the change of esthetic concepts. Because of the love for beauty and the unremitting pursuit of beauty in society, those female college students who are physically fit and who love beauty are more able to integrate into the society and are loved by people (Westfall et al., 2019).

Studies have shown that female college students with high levels of physical self-esteem have superior outcomes that make them happy, mentally healthy, and proud of themselves (Mathes and Kahn, 1975). At the same time, the lack of physical attractiveness is one of the main reasons for “leftover women,” so improving their body shape and physical self-esteem is one of the effective ways to improve the happiness of female college students. However, due to the lack of diet control, lack of exercise, learning pressure, and other reasons, more and more female college students are obese, and their body shape is not attractive, which then affects their original self-awareness, especially the body self-awareness. Due to the low level of physical self-esteem, many female college students blindly lose weight to improve their physical attractiveness and fail to establish a correct concept of health, resulting in a great negative impact on their body and mind. Therefore, how to improve the physical and mental health of female college students and improve their physical self-esteem has become an important subject for people to study.

Mental health refers to the various aspects of psychology and activity process in a good or normal state (Baidu Baike, 2022). The ideal mental health state is to maintain perfect personality, normal intelligence, correct cognition, appropriate emotion, reasonable will, positive attitude, appropriate behavior, and good adaptation (Hu, 2017). In recent years, the physique of the young students in our country continues to decline, and the rate of obesity and myopia remains high. The health problems of the students have attracted extensive attention from the education authorities and scholars. With the rapid development of society and the continuous improvement of living standards, as the national fitness has become a national strategy, people’s health awareness is gradually enhanced, especially female college students’ desire, and pursuit of health and beauty is becoming a new social trend and value orientation. However, due to environmental and life reasons such as high academic pressure, bad habits of work and rest, and lack of exercise, most female college students have different degrees of psychological disorders, which affect their healthy growth and quality of life. Therefore, how to improve the mental health of female college students, improve their life quality and mental health level has become an important subject for people to study (Zhang, 2000).

Cheerleading is a popular sport, collective gymnastics, skills, dance, music, fitness, and entertainment in one of the sports (Wu, 2009). Cheerleading originated in the United States and is one of the fastest-growing sports in the United States. Cheerleading members play an undeniable role in shaping the athletic image of the university, and they influence teenagers and young adults in a positive way (Johannes and Tucker, 2016). Under the accompaniment of music, practitioners through the expression of body movements and technical skills display, focusing on the youthful vitality, healthy and upward, the courage to take responsibility for the team spirit, with high fitness value, educational value, performance value, and humanistic value, at present in the country’s schools have been rapidly developed (Zhou, 2011). Cheerleading is divided into two main categories, namely, skill cheerleading and dance cheerleading. Skill cheerleading is accomplished through cheer, partner stunts, pyramid, basket toss, writhe, jump, and other movement techniques as the basic content, with music, clothing, team shape change, and marking items (such as pom, slogan board, horn, and flag), and other elements as the form of expression (Lu, 2011). The movement of dance cheerleading is smooth and generous, and the dynamic and passionate dance is full of unique personality, including pom, jazz, hip-hop, high kick, and free dance. Cheerleading exercise can not only effectively improve the physical quality of students, enhance the students’ physique but also on the student’s body of the system of the function and good psychological quality and temperament self-confidence exercise effect is very obvious. Therefore, female college students often participate in cheer exercise not only to shape a good body and improve the attractiveness of the body but also to improve their physical and mental health.

In recent years, cheerleading has become a fashionable sport favored by young students in China, especially by female college students. However, there are few studies on the influence of cheerleading on the level of physical self-esteem and mental health of female college students, and no systematic and in-depth theoretical system has been formed. Therefore, this article intends to investigate the changes in female college students’ physical self-esteem and mental health engaged in cheerleading exercise and explore the effective means to improve female college students’ physical self-esteem and mental health. This will be beneficial to female college students to improve their own physical self-esteem and mental health level and to broaden the relevant research field, for further digging cheerleading physical and mental development value of reference.

## Materials and Methods

We selected 76 female college students who participated in the amateur cheerleading training at the University of Electronic Science and Technology of China as the research objects. They were ordinary students from freshmen to seniors in various colleges, with an average age of 19.6 ± 0.7 years. Inclusion criteria were female college students who passed the physical examination and filled in the questionnaire, met the psychological symptoms, knew the study, and signed the informed consent. Exclusion criteria were patients with organ dysfunction, severe mental disorder, obvious physical injury or movement disorder, severe trauma, and operation history in recent 6 months, and those who are taking medication. Withdrawal criteria were those who are seriously injured or suddenly sick during the experiment requiring termination, and those who voluntarily request withdrawal. At the experimental stage, 13 subjects dropped out. Sixteen weeks later, the same scale was used to retest the psychological symptoms of the remaining 63 subjects. Sixty-three questionnaires were issued, and 63 valid questionnaires were collected, with an effective recovery rate of 100%.

Under the guidance of the special cheerleading coach (the author of this article), from March 2019 to June 2019, 63 female college students were trained in cheerleading for 16 weeks (4 times/week, 90 min/time). On Monday and Wednesday afternoon, we practice pom cheerleading, and on Friday and Sunday afternoon, we practice skill cheerleading. Every training includes 5 min activities preparation (from head to toe unarmed stretching exercise), 10 min warm-up (cheerleading hand a string of intermittent movement and jumps), 65 min the basic content of practice (including the cheerleading team learning, improve, strengthen, and consolidate the stage of practice content), 5 min of relaxation movement (in the context of soothing music, stretch, and massage the whole body to relax), and 5 min summary (including summing up the practice situation of each training, arranging the content of the next training, and the requirements of independent practice at ordinary times). The first to second week is the basic period of movement learning, and the main content is the basic form and the basic hand position of pom, the basic jumping movement, and the basic combination. The third to sixth week is the improvement period, and the main content is pom routine, skill level 1–2 routine, physical training, physical fitness training, and performance training. The 7th–12th week is the strengthening period, and the main contents are basic skills and physical training, competition routine and difficult movement training, physical fitness training, and performance training. The 13th–16th week is the consolidation period, the movement automation period, and the competition period. The main contents are skilled sets, standard basic movements, strengthening physical training, expressive training, highlighting personality style training, psychological endurance training, and accumulation of competition experience. The maximum exercise intensity is limited to 220 minus age, beyond which the intensity, interval, speed, difficulty, and complexity of the exercise may be adjusted. During the period of the whole experiment, the experimental personnel need to keep communication with students, keep weekly interview record of their life situations, ensure their cheerleading practice four times a week, and ensure students are less or no longer engaged in regular physical activity, but not including their private cheerleading practice, and maintain their old habits of everyday life.

Individual’s evaluation of physical self-esteem is affected by internal factors such as physical attraction, physical condition, physical quality, athletic ability, and body self-worth. The Chinese version of Physical Self-Perception Profile (PSPP) compiled by Fox and revised by Xu Xia was used to measure the physical self-esteem of 63 female college students. The scale consisted of one main scale and four subscales. The reliability and validity test of the scale showed that the krumbach coefficients of the main scale and the subscales ranged from 0.72 to 0.84 (Xu and Yao, 2001). This indicates that the physical self-esteem scale has good reliability and validity. Seventy-six female college students who participated in the amateur cheerleading training were investigated by using the PSPP at 0 and 16 weeks. The effective recovery rate of the two questionnaires was 100%.

The self-perception of somatization, obsessive-compulsive symptoms, interpersonal sensitivity, depression, anxiety, hostility, phobia, paranoia, psychosis, sleep and eating disorders in the recent one week were investigated by using SCL-90, widely used in clinical practice, at 0 and 16 weeks respectively (Jin et al., 2012), two questionnaires effective recovery rate was 100%. Through the statistical analysis of the questionnaire data of 0 weeks, it was found that the score of the total symptom index (total score divided by 90) of the self-rating scale of psychological symptoms of female college students was 33 students in the range of 1.5–2.5 (mild), 27 students in the range of 2.5–3.5 (moderate), 9 students in the range of 3.54.5 (heavy), and 7 students in the range of 4.5–5 (severe), which is the initial data before the experiment.

Excel software was used to calculate the score of physical self-esteem test, total score of psychological symptom test, and score of each factor of the subjects at 0 and 16 weeks. The total symptom index, mean score of positive symptom, and T score of each factor were calculated. Then, SPSS 19.0 was used for paired *T*-test analysis of relevant data, with significant level α = 0.05.

## Results

### Statistical Analysis of Scores in Each Sub-Area of Physical Self-Esteem Before and After the Experiment

Through the mean value, standard deviation, and *T*-test of the score of the physical self-esteem questionnaire before and after the experiment, the results showed that in the amateur cheerleading training, the score of the body condition subscale before the experiment was the highest, which was 16.75 ± 2.15. The lowest score of the body self-worth scale was 14.96 ± 2.55. After the experiment, the score of the physical condition subscale was still the highest (17.98 ± 1.52). The motor ability subscale was the lowest with a score of 16.31 ± 3.43. The paired *T*-test results showed that after cheerleading training, the subjects had significant differences in physical self-esteem before and after the experiment, among which the *T*-value of the main scale of physical self-worth was 3.35, the physical fitness was 4.39, and the physical attractiveness was 4.18. The *P*-value of several scales was all less than 0.01. The *T*-value of the exercise ability subscale was 2.88, and the *T*-value of the physical condition subscale was 3.87, indicating a significant difference between them (*P* < 0.05). These results indicate that cheerleading training can effectively improve female college students’ physical self-esteem, especially in the aspect of physical attractiveness, and effectively improve the level of self-worth in the main areas (Deng, 2010).

The results of horizontal variance analysis of each subfield of female college students’ physical self-esteem before and after the experiment showed that there were significant differences in the self-worth of the main scale, physical attraction, and physical quality of the subscale before and after the experiment (*P* < 0.01), and significant differences in the physical condition and athletic ability (*P* < 0.05) (see Table 1).

**TABLE 1:** *T*-test results of subjects’ scores in each sub-area of physical self-esteem before and after the experiment.

	0 Weeks	16 Weeks	*t*	*P*
Self-worth	14.96 ± 2.55	16.43 ± 2.07	3.35	0.001
Sports ability	15.95 2.73	16.31 ± 3.43	2.88	0.023
Physical condition	16.75 ± 2.15	17.98 ± 1.52	3.87	0.039
Physical attraction	15.15 ± 2.50	17.72 ± 2.18	4.18	0.000
Physical quality	14.99 ± 2.37	16.47 ± 1.04	4.39	0.006

### Statistical Analysis of Scores of Each Factor of Psychological Symptoms Before and After the Experiment

According to the mean and standard deviation of *T*-score of each factor of SCL-90 before and after the experiment, the scores of each factor of the psychological symptom of 63 female college students before the experiment were somatization (1.62 ± 0.87), psychosis (1.76 ± 0.86), sleep and eating disorder (1.79 ± 0.85), terror (1.80 ± 0.93), paranoid (1.85 ± 0.83), depression (1.88 ± 0.92), anxiety (1.90 ± 0.88), sensitive interpersonal relationship (1.96 ± 0.83), hostility (1.97 ± 0.98), and forced symptoms (2.10 ± 0.97). After 16 weeks of the experiment, the scores of each factor from low to high were somatization (1.48 ± 0.61), psychosis (1.52 ± 0.60), sleep and eating disorders (1.57 ± 0.57), horror (1.57 ± 0.63), paranoid (1.60 ± 0.57), anxiety (1.64 ± 0.59), depression (1.67 ± 0.62), hostile (1.67 ± 0.66), sensitive interpersonal relationship (1.70 ± 0.53), and forced symptoms (1.79 ± 0.62). From the comparison of the mean difference of the factors before and after the experiment, it was found that the improvement effect of cheerleading on the psychological factors of female college students was somatic, depression, sleep and eating disorders, terror, paranoia, psychosis, interpersonal sensitivity, anxiety, hostility, and compulsion symptoms.

Paired *T*-test results of each factor score before and after the experiment showed that the subjects before and after the experiment were somatization (*t* = 6.485), obsessive-compulsive symptoms (*t* = 11.716), interpersonal sensitivity (*t* = 10.077), depression (*t* = 8.403), anxiety (*t* = 10.767), hostility (*t* = 10.866), terror (*t* = 9.260), paranoid (*t* = 10.414), psychosis (*t* = 9.610), sleep and eating disorders (*t* = 9.323), and total symptom index (*t* = 13.245). The mean score of positive symptoms (*t* = 12.237) was significantly different (*P* < 0.01) (see Table 2).

**TABLE 2:** *T*-test results of all factors, total symptom index, and positive symptom of SCL-90 before and after the experiment.

		Difference in pairs					*t*	df	Sig. (double side)
		Mean	Standard deviation	Standard error of the mean	95% confidence interval for difference				
					Lower limit	Ceiling			
Somatization	Before–After	0.14137	0.31212	0.02180	0.09838	0.18435	6.485	62	0.000
Forced symptoms	Before–After	0.31561	0.38571	0.02694	0.26249	0.36873	11.716	62	0.000
Interpersonal sensitivity	Before–After	0.25868	0.36755	0.02567	0.20807	0.30930	10.077	62	0.000
Depression	Before–After	0.21244	0.36199	0.02528	0.16259	0.26229	8.403	62	0.000
Anxiety	Before–After	0.25951	0.34509	0.02410	0.21199	0.30703	10.767	62	0.000
Hostile	Before–After	0.30141	0.39718	0.02774	0.24672	0.35611	10.866	62	0.000
Terrorist	Before–After	0.23722	0.36678	0.02562	0.18671	0.28773	9.260	62	0.000
Paranoid	Before–After	0.24288	0.33392	0.02332	0.19689	0.28886	10.414	62	0.000
Psychotic	Before–After	0.24293	0.36193	0.02528	0.19309	0.29277	9.610	62	0.000
Sleep and eating disorders	Before–After	0.22493	0.34542	0.02412	0.17736	0.27249	9.323	62	0.000
Overall symptom index	Before–After	0.29317	0.31693	0.02214	0.24953	0.33681	13.245	62	0.000
Positive symptoms were equally divided	Before–After	0.27693	0.32402	0.02263	0.23231	0.32155	12.237	62	0.000

## Discussion

According to the analysis of the physical self-esteem and mental health of female college students before and after the cheerleading exercise experiment, it is found that there are significant differences between cheerleading exercise and their physical self-esteem and mental health level. This is because long-term cheerleading exercises can not only eliminate the excess fat of the exerciser, make muscle with renewed vigor and enhance the flexibility of the muscle, create a strong and handsome tall and straight posture and body shape, but also to rectify the shoulders, chest, neck and legs, bad posture, at the same time can improve cardiopulmonary function, enhance physical fitness. Physical health can effectively improve physical self-esteem and improve the body system discomfort, anxiety, and other manifestations of physical discomfort (Phanudulkitti et al., 2016).

### Influence of Cheerleading Exercise on Female College Students’ Physical Self-Esteem

First of all, cheerleading has the special effect of shaping the beauty of female college students. The style and rhythm of dance cheerleading vary from dance to dance, but dancers must maintain good posture: chest out, stomach in, waist up, shoulder lock, butt head, hips clamped up, and muscle coordination (Xie, 2011). This beautiful, dignified, elegant modeling not only gives people a sense of vitality but also contains a great artistic charm, beautiful posture, modeling, and theme that are fully reflected. Cheerleading has strict requirements on footsteps, posture, balance, strength, and control and has very delicate requirements on rhythm, orientation, upper limb movements, rotation, and other techniques. Long-term cheerleading amateur training can eliminate excess body fat, make muscles revitalize, and enhance muscle elasticity, shaping a straight and handsome body posture and body shape, and also conducive to correct shoulder, chest, neck, and leg bad posture. These effects and effects of cheerleading will improve the level of physical self-esteem of exercisers, especially in respect of physical attractiveness.

Second, cheerleading is beneficial to improve the physical quality of female college students. Cheerleading enthusiastic unrestrained movement, rhythm, and speed change is complex; the movement load is larger; and it has certain requirements for the human head, shoulder, waist, hip, leg, muscle, and joint movement. In cheerleading exercise, the practitioner should carry on the wonderful movement with different rhythms; in the 2 min or so, the routine will do multiple movements per second on average. Therefore, regular participation in the right amount of cheerleading exercise can enhance physical fitness and effectively improve the strength of the human body, speed, bouncing, coordination, agility, and other physical qualities. The physical quality and the sports ability self-esteem level of the female college students have significantly improved.

Third, cheerleading is beneficial to the health of heart and lung function of female college students. During cheerleading exercises, you can dance as much as you like. Good exercise effect can be achieved in moving and static, fast and slow, coordination and cooperation, rotating and jumping, stunt and toss, rolling and tumbling, etc. This can not only effectively improve the aerobic metabolism of female college students, but also enhance their physiological function and improve their cardiopulmonary function, and effectively improve their physical condition self-esteem level.

Fourth, cheerleading is beneficial to the social and entertainment of female college students. Comfortable and relaxed socializing is one of the personal needs. Cheerleading can be an important social tool, and participating in it can promote communication between people and improve communication ability, so that participants become better at interpersonal communication, people, public relations, social, unity, and cooperation advantage. Facing the great pressure of study, female college students are often in a state of tension and worry, but in the melodious and passionate music, showing a section of cheerleading, students can get a psychological adjustment and maintain a good state of mind and happy mood, so as to improve the self-esteem level of self-worth.

Fifth, cheerleading is good for improving the self-confidence of female college students. Cheerleading team pays great attention to unity and cooperation, with the aim of pursuing the highest honor of the team. Under the team culture of team motivation and mutual help, the cheerleading team creates an atmosphere of support, encouragement, and cooperation for female college students. The self-efficacy of female college students is increased, the value is reflected, and it helps to improve their confidence (Kao and Watson, 2014). At the same time, cheerleading training pays special attention to the athlete’s shape and temperament training, the competition, and the performance need of athletes through body movements, emotions, expressions, props, and others that fully show themselves to the audience to pass the positive energy spirit and sports beauty, which helps improve the confidence of female college students.

### Influence of Cheerleading Exercise on the Mental Health of Female College Students

Dauwan et al. (2019) found that physical exercise was superior to conventional treatment in improving the quality of life, depressive symptoms, cognitive domains of attention and working memory, executive ability, and spirit. The evidence supporting exercise as a treatment for depression is compelling, but what kind of exercise, amount of exercise, frequency, and intensity are necessary to make a significant difference (Amrita et al., 2018). Early adolescents with more severe depression may see greater improvement with preferred music therapy (Chen et al., 2019). In this study, we suggest that the combination of exercise and music may have a more beneficial effect on the treatment of depression than exercise or music alone. Melodious and passionate music show a humorous, playful, cool, and exaggerated hip-hop; carry out a touching, moving, beautiful jazz; jump a dynamic, passionate, exciting freestyle pom; and even practice some difficult skills cheerleading, and these can let the practitioners temporarily get rid of meaningless thoughts, impulses, and behaviors over time through the transfer of attention in the treatment of compulsive symptoms that have a certain positive role. In addition, the combination of beautiful music and dynamic cheerleading is a kind of beautiful enjoyment for the visual, auditory, and kinesthetic of the practitioners, which can make the practitioners feel happy and refreshed, reduce bad mood, and effectively relieve depression symptoms.

Sports can enhance the communication between people, and it is an important means to promote people’s friendship and strengthen unity (Wu, 2022). A study suggests that incorporating psychotherapy into exercise is more likely to improve interpersonal sensitivity, develop a healthy personality, and maintain a stable mood (Zhang and Zheng, 2004). Our results are consistent with that. Female college students in the same environment for cheerleading exercise together, especially when practicing skills cheerleading team coordination, it expanded in the process of movement of emotional communication, enhance mutual understanding, has realized the exchanges and set up specific relationships, such as people coordination and interactive performance, eye contact, group display, slowly each other harmonious, mutual trust and positive interaction, communication and exchanges, gradually establish emotional foundation and humanistic spirit is beneficial to improve female college students’ sensitive tendency of interpersonal relationship, cultivate healthy personality and stable mood, and establish friendly thoughts, emotions and behaviors (Zhang et al., 2005).

From the point of view of psychology, terror is a kind of strongly depressed emotional experience produced by human or animals when they are faced with a dangerous situation or are worried about some danger or fear, and try to get rid of it without any ability or potential consciousness (Cai, 2017), such as afraid of taking transportation to travel, open space fear, crowd or public fear, social terror, and stage terror (Wang and Yao, 2005). In the cheerleading exercise, team communication, individual display, collective performance, stage experience, the accumulation of games, happy environment, etc., can help individuals to build confidence, improve the mood, and overcome the terror.

At present, there are few studies on the influence of physical exercise on hostility and paranoia, and it is more difficult to find the research results of cheerleading exercise in this aspect. However, we can find reference results from relevant studies. For example, Nouri selected 100 male subjects from a university in the Midwest of the United States and divided them into five groups, namely, high intensity, moderate intensity, primary intensity, jogging dropout, and non-exercisers (Nouri and Beer, 1989). They were assessed on the Commitment to Running Scale, the Bus-Durkee Inventory, which measures hostility and aggression, and the State-Trait Anxiety Scale data analysis showed that the average aggression and hostility scores of non-exercisers were higher than those of dropouts or joggers. There was no significant difference between dropouts and advanced joggers, but their scores were significantly lower than those of other joggers, confirming that jogging had a positive effect on hostility and aggression. This study argues that fair cheerleading competitions and standard action requirements can teach students to stop being paranoid and hostile. During the cheerleading exercise, practitioners must keep good hold out a bosom, closed abdomen, waist, locked out, stem head, hip and locked position, the muscles, joints, limbs, action and skill, the coordination between the center of gravity of practitioners, balance, power, control, breathing, rhythm, azimuth, rotation, and other technology have very sensitive demand, also to the human body the head, shoulder, waist, leg, muscle, and joint movement has certain norms. The competition needs to respect the opponent, the referee, and the rules. The success or failure of the competition can temper the will quality. These constraints, requirements, and experience can subtly adjust the hostile and paranoid psychology of female college students.

Sleep disorders may cause depression, anxiety, hostility, and other mental diseases, so improving the sleep quality can promote the physical and mental health of college students (Herring et al., 2015). Exercise as a means to improve the body’s sleep quality has been recognized by the majority of scholars (Yang et al., 2018). Cheerleading sports can promote the sleep of female college students and improve their sleep quality. This is because the cheerleading exercises constantly stimulate the various needs of the body, such as increased oxygen uptake, circulation system, more suitable muscle strength, and range increased, energy consumption increase, good mood, and good appetite, which can adjust the bad mood, improve the anxiety, effectively improve sleep disturbances, and enhance the positive effect of dietary changes.

Psychosis reflects a wide range of acute symptoms and behaviors (Hu et al., 2013). Cheerleading exercise can improve the abovementioned aspects of different levels of psychological symptoms, such as improving individual somatization, sleep, depression, terror, and paranoia; these psychological symptoms after adjustment can reduce the loneliness of female college students, establish the sense of belonging, and enhance the affinity and happiness, so as to improve the symptoms of psychotic behavior.

All in all, the factors of psychological symptoms do not exist in isolation, but interact with each other and restrict each other. Improvement of one factor may affect other factors. Cheerleading exercise has played a positive role in improving the psychological symptoms and the overall level of mental health of female college students.

## Conclusion

After 16 weeks of cheerleading exercise, there was a significant difference in the level of physical self-esteem of female college students. It shows that cheerleading exercise with a certain intensity, duration, and frequency can affect and improve female college students’ physical condition, physical quality, athletic ability, and self-esteem level of body self-worth, especially the self-esteem level of body attraction. Among them, the self-esteem levels of physical attractiveness, physical quality, and physical self-worth were improved to the greatest extent, while the self-esteem levels of athletic ability and physical condition were significantly improved. This is because cheerleading has the special effect of shaping the beauty of female college students, it is beneficial to improve the body quality of female college students, promote their cardiopulmonary function, enhance their social skills, and provide the entertainment elements, which directly or indirectly improve the female college students in physical attractiveness, physical quality, health, and sports ability levels of self-esteem and self-worth (Deng and Yu, 2018). In future studies, the differences in different grades, family background, technical level, and mental health status can be fully considered in the selection of subjects, so as to fully guarantee the control conditions of certain intensity, duration, and frequency and overcome the deficiencies in methodology to design a more standardized and complete experiment and further explore the influence value of cheerleading on female college students’ physical self-esteem. According to the conclusion of this study and previous research results, female college students should take part in cheerleading through various ways to fully understand and better improve their physical self-esteem. At the same time, the exercise value of cheerleading is also very suitable for young children of different ages to participate in, and we hope that the school can carry out and popularize cheerleading.

After 16 weeks of cheerleading exercise, there were significant differences in somatization, obsessive-compulsive symptoms, interpersonal sensitivity, depression, anxiety, hostility, terror, paranoia, psychosis, sleep and eating disorders, total symptom index, and positive symptoms before and after the experiment. Cheerleading has the special effect of shaping the beauty of female college students, which is beneficial to improve their physical quality, promote the health of their heart and lung function, establish their courage and self-confidence, enhance their social ability and provide them with entertainment elements. Which, directly or indirectly, to improve the female college students in somatization, force symptom, interpersonal sensitivity, depression, anxiety, hostility, terror, paranoia, psychosis, sleep and eating disorders of adverse symptoms, especially obvious improvement of somatization, depression, sleep and eating disorders, thus effectively improve the overall level of mental health. Cheerleading exercise can be used as one of the means of prevention and treatment of psychological disorders. According to the conclusion of this study and previous research results, the exercise value of cheerleading is very suitable for different age groups to participate in, and female college students should participate in cheerleading through various ways, fully understand, and better improve their mental health level. Since there are many factors causing psychological disorders in female college students, it is hoped that future research can be combined with family environment, academic pressure, love status, regional differences, seasonal climate, and other factors to carry out a comprehensive study. At the same time, we should explore more effective sports means and methods to improve the mental health level of female college students and further enrich the value theory of sports.

## Data Availability Statement

The original contributions presented in this study are included in the article/supplementary material, further inquiries can be directed to the corresponding author.

## Ethics Statement

Ethical review and approval were not required for the study on human participants in accordance with the local legislation and institutional requirements. The patients/participants provided their written informed consent to participate in this study.

## Author Contributions

CD was responsible for the cheerleading experiment and data analysis and drafted and revised this manuscript. QY, GL, and SL participated in the exchange and discussion and provided suggestions on the experimental design. All authors contributed to the article and approved the submitted version.

## Conflict of Interest

The authors declare that the research was conducted in the absence of any commercial or financial relationships that could be construed as a potential conflict of interest.

## Publisher’s Note

All claims expressed in this article are solely those of the authors and do not necessarily represent those of their affiliated organizations, or those of the publisher, the editors and the reviewers. Any product that may be evaluated in this article, or claim that may be made by its manufacturer, is not guaranteed or endorsed by the publisher.
